# Cluster randomized trial comparing standard versus enhanced implementation strategies for improving outreach to persons with SMI: 12-month results

**DOI:** 10.1186/1748-5908-10-S1-A26

**Published:** 2015-08-20

**Authors:** David E Goodrich, Daniel Almirall, Kristin M Abraham, Kristina M Nord, Zongshan Lai, Nicholas W Bowersox, Amy M Kilbourne

**Affiliations:** 1 VA Center for Clinical Management Research, VA Ann Arbor Healthcare System, Ann Arbor, MI, 48105, USA; 2 Department of Psychiatry, University of Michigan Medical School, Ann Arbor, MI, 49109-2800, USA; 3 Institute for Social Research, University of Michigan, Ann Arbor, MI 48104-2321, USA; 4 School of Public Health, University of Michigan, Ann Arbor, MI 48109-5425, USA; 5 University of Detroit Mercy, Detroit, MI 48221-3038, USA

## Objective

This study compared the effectiveness of an enhanced versus standard implementation strategy (Replicating Effective Programs-REP) for providers at VA outpatient facilities on improving uptake of a national outreach program for Veterans with serious mental illness (Re-Engage) among sites not initially responding to a standard implementation strategy.

## Methods

Initially, Re-Engage was implemented at 158 VA facilities by mental health providers who received the standard REP strategy to support uptake (implementation manual, training, and technical assistance). Re-Engage involved giving providers a list of patients with serious mental illness who had not been seen at their facility for at least a year, requesting that providers contact these patients, assess their clinical status, and where appropriate, expedite VA healthcare appointments. At month 6, facilities considered non-responsive(N = 88, total of 3,200 patients), defined as <80% of patients on providers' lists with updated assessment of clinical status, were randomized to receive either Enhanced REP (REP+Facilitation; N = 39 practices) for 6 months followed by standard REP for 6 months; or continued standard REP (N = 49 practices) for 6 months followed by 6 months of Enhanced REP for facilities still not responding. Enhanced REP consisted of monthly phone-based coaching by national experts in Re-Engage on overcoming adoption barriers. Quantitative outcomes included attempted contacts and subsequent receipt of outpatient care.

## Results

Patients from facilities randomized to receive Enhanced compared to standard REP were more likely to have an attempted contact (30% vs. 13%, p < .001). Sites that received Enhanced REP six months after randomization (delayed implementation of Facilitation) were no more likely to have increased contacts. There were no differences in patient-level utilization between Enhanced and standard REP sites 12 months post-randomization.

## Implications

Adaptive implementation intervention strategies like Enhanced REP when applied immediately to address implementation non-response, offer a means to augment implementation efforts.

## Funding Source

VA HSR&D (SDR 11-232).

